# Child–Adult Transition in Sarcoidosis: A Series of 52 Patients

**DOI:** 10.3390/jcm9072097

**Published:** 2020-07-03

**Authors:** Simon Chauveau, Florence Jeny, Marie-Emeline Montagne, Rola Abou Taam, Véronique Houdouin, Ulrich Meinzer, Christophe Delacourt, Ralph Epaud, Fleur Cohen Aubart, Catherine Chapelon-Abric, Dominique Israël-Biet, Karine Juvin, Antoine Dossier, Bahram Bodaghi, Grégoire Prévot, Jean-Marc Naccache, Sarah Mattioni, Antoine Deschildre, Jacques Brouard, Abdellatif Tazi, Roderich Meckenstock, Morgane Didier, Julien Haroche, Annick Clement, Jean-François Bernaudin, Hilario Nunes, Dominique Valeyre, Nadia Nathan, for the French Sarcoidosis Group (GSF)

**Affiliations:** 1 AP-HP Pulmonology Department, Avicenne Hospital, 93000 Bobigny, France; simon.chauveau@aphp.fr (S.C.); florence.jeny@aphp.fr (F.J.); morgane.didier@aphp.fr (M.D.); jean-francois.bernaudin@aphp.fr (J.-F.B.); hilario.nunes@aphp.fr (H.N.); dominique.valeyre@aphp.fr (D.V.); 2 Inserm UMR_1272, Laboratory of Hypoxia & Lung Université Sorbonne Paris Nord, 93000 Bobigny, France; 3 AP-HP Pediatric Pulmonology Department and Reference Center for Rare Lung Diseases (RespiRare), Armand Trousseau Hospital, 75012 Paris, France; montagnemarieemeline@gmail.com (M.-E.M.); annick.clement@aphp.fr (A.C.); 4 AP-HP Pediatric Pulmonology Department and Reference Center for Rare Lung Diseases (RespiRare), Necker Enfants Malades Hospital, 75015 Paris, France; rola.aboutaam@aphp.fr (R.A.T.); christophe.delacourt@aphp.fr (C.D.); 5 AP-HP Pediatric Pulmonology Department, Robert Debré Hospital, 75019 Paris, France; veronique.houdouin@aphp.fr; 6 AP-HP Department of General Pediatrics, Pediatric Internal Medicine, Rheumatology and Infectious Diseases, National Referee Center for Rare Pediatric Inflammatory Rheumatisms and Systemic Auto-Immune Diseases RAISE, Robert Debré University Hospital, 75019 Paris, France; ulrich.meinzer@aphp.fr; 7 Pediatric Department and Reference Center for Rare Lung Diseases (RespiRare), Centre Hospitalier Intercommunal de Créteil, 94000 Créteil, France; ralph.epaud@chicreteil.fr; 8 University Paris Est Creteil, INSERM, IMRB, F-94010 Creteil, France; 9 Sorbonne Université, Assistance Publique Hôpitaux de Paris, Hôpital de la Pitié-Salpêtrière, Service de Médecine Interne 2, French National Reference Center for rare Systemic diseases, 75013-Paris, France; fleur.cohen@aphp.fr (F.C.A.); julien.haroche@aphp.fr (J.H.); 10 AP-HP Department of Internal Medicine and Clinical Immunology, La Pitié Salpétrière Hospital, AP-HP, 75013 Paris, France; catherine.chapelon@aphp.fr; 11 AP-HP Pulmonology Department, HEGP Hospital, 75015 Paris, France; dominique.israel-biet@aphp.fr (D.I.-B.); karine.juvin@aphp.fr (K.J.); 12 AP-HP Department of Internal Medicine, Bichat Hospital, 75018 Paris, France; antoine.dossier@aphp.fr; 13 AP-HP Department of Ophtalmology, La Pitié Salpétrière Hospital, IHU FOReSIGHT, Sorbonne Université, 75013 Paris, France; bahram.bodaghi@aphp.fr; 14 AP-HP Department of Ophtalmology, Avicenne Hospital, IHU FOReSIGHT, 93000 Bobigny, France; 15 Pulmonology Department, Larrey University Hospital, 31400 Toulouse, France; prevot.g@chu-toulouse.fr; 16 AP-HP Pulmonology Department, Tenon Hospital, 75020 Paris, France; jean-marc.naccache@aphp.fr; 17 AP-HP Department of Internal Medicine, Tenon Hospital, 75020 Paris, France; sarah.mattioni@aphp.fr; 18 Paediatric Pulmonology and Allergy Unit, CHU Lille, Université de lille, Hôpital Jeanne de Flandre, F-59000 Lille, France; antoine.deschildre@chru-lille.fr; 19 Pediatric Pulmonology Department, Caen University Hospital, 14000 Caen, France; brouard-j@chu-caen.fr; 20 AP-HP Pulmonology Department, Saint Louis Hospital, 75010 Paris, France; abdellatif.tazi@aphp.fr; 21 Internal Medicine and Infectious Diseases Department, Versailles Hospital, 78150 Le Chesnay, France; rmeckenstock@ch-versailles.fr; 22 Inserm UMR-S933, Childhood Genetic Disorders, Sorbonne Université, 75012 Paris, France

**Keywords:** sarcoidosis, children, transition of care, interstitial lung disease

## Abstract

(1) Background: Pediatric sarcoidosis is a rare and mostly severe disease. Very few pediatric series with a prolonged follow-up are reported. We aimed to evaluate the evolution of pediatric sarcoidosis in adulthood. (2) Material and methods: Patients over 18-years-old with a pediatric-onset sarcoidosis (≤15-year-old) who completed at least a three-year follow-up in French expert centers were included. Clinical information at presentation and outcome in adulthood were studied. (3) Results: A total of 52 patients were included (34 prospectively in childhood and 18 retrospectively in adulthood), with a mean age of 12 (±2.7) at diagnosis. The median duration time of follow-up was 11.5 years (range 3–44.5). Relapses mostly occurred during treatment decrease (84.5%), others within the three years after treatment interruption (9.1%), and rarely when the disease was stable for more than three years (6.4%). Sarcoidosis was severe in 11 (21.2%) in adulthood. Patients received a high corticosteroid cumulative dose (median 17,900 mg) for a median duration of five years (range 0–32), resulting in mostly mild (18; 35.3%) and rarely severe (2; 3.8%) adverse events. (4) Conclusions: Pediatric-onset sarcoidosis needed a long-term treatment in almost half of the patients. Around one fifth of pediatric-onset sarcoidosis patients had severe sarcoidosis consequences in adulthood.

## 1. Introduction

Sarcoidosis is a systemic granulomatous disease of unknown origin assumed to be multifactorial, associating a genetic susceptibility and an environmental exposure, mineral or organic, that triggers or enhances the inflammatory and granulomatous process [1,2]. Sarcoidosis epidemiology is characterized by wide variations according to age, sex, ethnicity, geographical origin, environment, socioeconomic status, and even across time [3]. It has been shown that sarcoidosis onset is diagnosed at a later age in recent times, with an increasing percent of people aged >45, respectively 80% in females and 44% in males, as reported in a recent study [4]. Sarcoidosis is far less common in children than in adults as shown in France where the incidence is respectively 4.9/100,000/years in adults and between 0.40 and 0.80/100,000/years in children [5,6]. Children of all ages can be affected, but more than two thirds of the patients are over 10 years old at the time of diagnosis [6]. Interestingly, there are striking differences in sarcoidosis presentation between children and adults, with general symptoms being more frequent in children, including fever (44%), weight loss (44%), and sweats (10%) as well as the extension of the disease with 46.6% of them having 4 or more organs involved (median 3.4 organs) at diagnosis compared to only 7% in adults [6,7,8,9]. Pediatric sarcoidosis evolution is poorly reported, with a highly heterogeneous follow-up duration and evaluation criteria [6,10,11,12,13].

Considering the differences between children and adult sarcoidosis, we found it interesting to focus on long-term evolution of children-onset sarcoidosis in adulthood. This was made easy thanks to national organizations: the French sarcoidosis group (GSF)—an official scientific working group of the Société de Pneumologie de Langue Française—which involves more than 100 physicians, including pediatric pulmonologists and researchers; and the French network for rare lung diseases (RespiFIL; https://respifil.fr), which promotes and organizes better care for patients with all kinds of rare lung diseases and offers comprehensive web-based multidisciplinary discussions for better care of rare lung diseases.

The aim of this study was to report, in a large national series of pediatric-onset sarcoidosis, the evolution of sarcoidosis in the patients that reached adulthood, and to identify if variables collected at disease onset can predict sarcoidosis severity and treatment status in adulthood.

## 2. Experimental Section

### 2.1. Patients

The study was observational, retrospective, and multicentric in France. A series of pediatric and adult patients who present(ed) a pediatric-onset sarcoidosis (i.e., before the age of 16) were eligible to be registered in the e-RespiRare database of the French reference center for rare lung diseases (RespiRare; www.respirare.fr), that is part of RespiFIL [14]. E-RespiRare has been created in 2008 and is an online nation-wide database used for clinical follow-up as well as for research purposes. The clinicians can register the patients by two different ways. For pediatric patients, files can be registered prospectively. On the other side, for other patients, the medical data can also be registered in the database afterwards, in adulthood, at any stage of the sarcoidosis evolution, with a retrospective collection of the pediatric data using available medical files. 

Patients included in the study were retrieved from the database and needed to meet diagnosis confirmed by files review by SC, MEM, FJ, NN, and DV. Among the patients with an ascertained diagnosis of pediatric-onset sarcoidosis [1], those who completed at least a three-year follow-up and reached at least the age of 18 were included. 

Information from the medical files at adult age was provided by the referring practitioners. For patients without any follow-up in the past three years, a phone consultation was proposed. If phone consultation was not possible, city registries were consulted to check whether the patient was still alive. 

### 2.2. Collected Data

The following clinical data were collected: age at diagnosis, gender, geographic ancestry, history of familial sarcoidosis, clinical presentation including severity for each affected organ respiratory function test when available: Forced vital capacity (FVC), forced expiratory volume in one second (FEV1), and DLCO, follow-up duration, treatments, disease evolution including sarcoidosis relapse or control or stability, and adverse events of treatment. Disease evolution was defined as “stable”, i.e., no relapse in more than one year after treatment discontinuation; “controlled”, i.e., no relapse within the past year in a patient under treatment; “uncontrolled” i.e., a relapse and/or requirement for an increase in treatment within the previous year; “lung transplantation” or “death”. Severe sarcoidosis was defined as: (i) lung involvement with Composite Physiologic index (CPI) > 40 and/or pulmonary hypertension and/or pulmonary fibrosis extent at CT of more than 20% of the lung [15]; (ii) evidence of central nervous system sarcoidosis requiring treatment; (iii) heart involvement with spontaneous sustained ventricular tachycardia, arrythmias requiring defibrillator, conduction block requiring pacemaker, heart failure with left ventricular ejection fraction under 40%; (iv) liver involvement requiring a transplantation; (v) kidney involvement requiring chronic dialysis or transplantation. We compared each organ involvement occurrence between sarcoidosis onset in childhood and adulthood.

### 2.3. Statistics

Statistical analyses were performed using GraphPad Prism version 6.0 and STATA (version13, stata corp, College station, TX, USA). Numerical data were tested for normality by D’Agostino K squared Test. Quantitative values were reported as mean and standard deviations (SD) if normality were assessed, otherwise as median and range. Qualitative data were reported as number (percentages). Comparisons of organ involvement between pediatric and adult patients, comparison between prospective pediatric-onset sarcoidosis and retrospective reported pediatric-onset sarcoidosis were established using a Wilcoxon matched-pairs signed rank test, Mann–Whitney test, Chi^2^, and Fisher’s exact test when appropriate. Association between variables at diagnosis onset, treatment status, and occurrence of severity were carried out with a Cox regression model. All tests were two sided. *p*-values less than 0.05 were considered statistically significant.

### 2.4. Legal Dispositions

The RespiRare database has been organized and structured as an electronic medical record for patients, allowing the storage of data (including clinical, biological, functional, and genetic information) through a web interface. It allows, after each center’s authorization, access to the patients’ data filtered through a central identification number for research purposes. The institutional ethics review boards approved the use of this database register (“Commission Nationale de l’Informatique et des Libertés”, CNIL no. 908324 and the “Comité Consultatif sur le Traitement de l’Information en matière de Recherche dans le domaine de la Santé”, CCTIRS no. 08.015bis). Patients and their families were informed of the nature and goal of all investigations and gave their informed consent.

## 3. Results

### 3.1. Study Population

**Figure 1:**
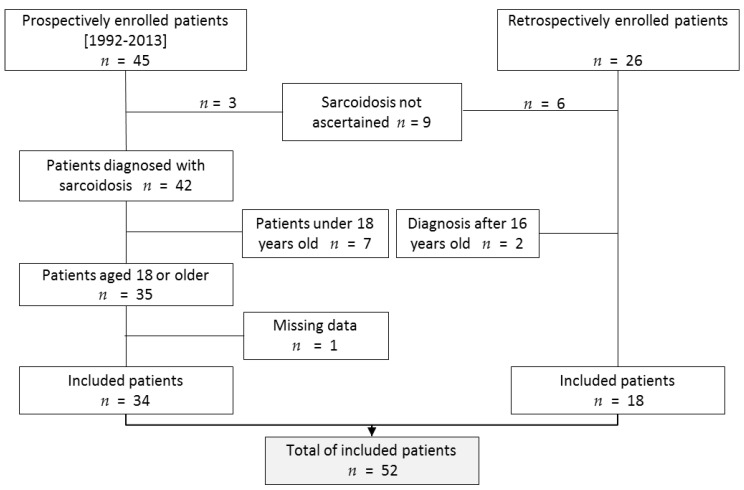
Flow chart of the study. A total of 71 patients were listed in the pediatric sarcoidosis database. Among them, 52 finally met the inclusion criteria.

A total of 71 patients with a proposed diagnosis of pediatric sarcoidosis were recorded in the RespiRare database. Among them, nine patients were excluded because the diagnosis criteria were not fulfilled, and two because age at onset was above 16. Seven extra patients were not included because they had not reached the age of 18 at the moment of the study (because they were still in pediatric follow-up). In one patient, no medical update was available at adulthood even though we could check she was still living thanks to the city registry. In total, 52 patients could be included (Figure 1). In 51 patients, diagnosis was assessed by histopathology of a biopsy demonstrating noncaseating epithelioid cell granulomas. In one patient, clinical feature, characteristic chest X-ray, lymphocytic alveolitis with a CD4+/CD8+ above 4 [16], and elevated serum angiotensin-converting enzyme substantiated the diagnosis.

The clinical characteristics of the 52 included patients are provided in Table 1. The female/male ratio was 1.26. A majority of patients (71.1%) had a Sub-Saharan Africa or Afro-Caribbean isles ancestry. The median duration of follow-up after the disease onset was 11.5 years, (range: 3–44.5).

As shown in Supplementary Table S1, there was no significant difference between the pediatric prospective group and the retrospective adult group, except length of follow up (9.8 years versus 17.8 years respectively, *p* = 0.005). (Supplementary Table S1). At the time of the study, 41 patients were still followed by an adult sarcoidosis expert (pulmonologist, internist, nephrologist, ophthalmologist, or hematologists) in eight different French university hospitals. The specialized follow-up had been discontinued for 11 patients; their evolution was assessed either by their general practitioners or by phone contact with the patient themself.

During follow-up, a few patients got comorbidities: two women presented Crohn’s disease diagnosed more than seven years after the diagnosis, and in three other patients, acquired haemophilia A, immune thrombocytopenia, or hemochromatosis were diagnosed more than ten years after the diagnosis.

### 3.2. Evolution from Childhood to Adulthood

#### 3.2.1. Disease Activity During Follow-Up

**Figure 2:**
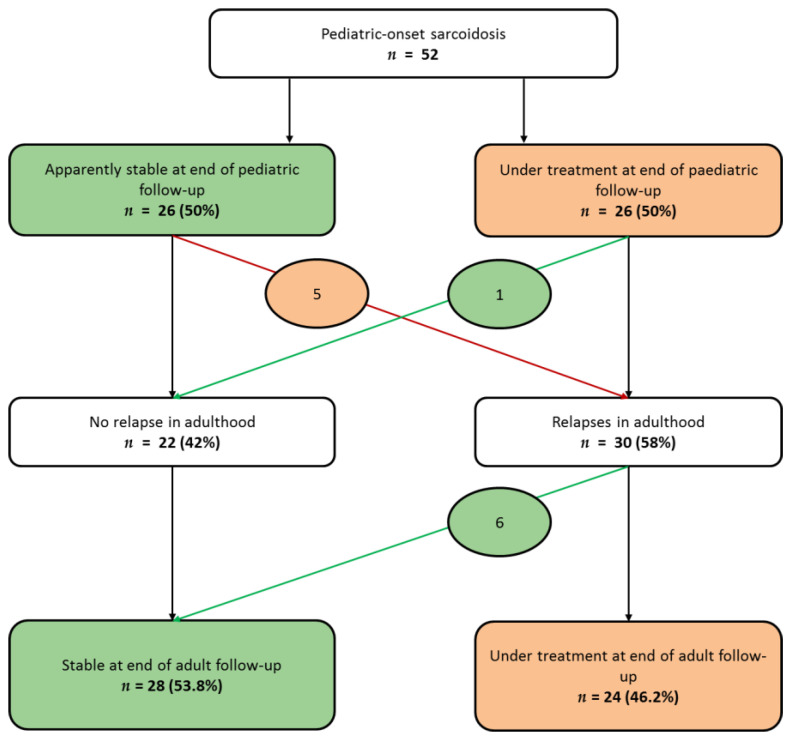
Sarcoidosis evolution from last pediatric evaluation to last adult evaluation. At the end of the pediatric evaluation of the 52 patients, half of them were stable, whereas the other half still required treatment. Patients whose evolution changed from “stable” to “under treatment” are circled in orange; patients whose evolution changed from “under treatment” to “stable” are circled in green. (Stable was defined as no relapse more than one year after treatment discontinuation).

As shown in Supplementary Figure S1, among the 34 patients prospectively registered in childhood, 18 (52%) were healed during childhood, with no relapse during the follow-up in adulthood (median duration 117 months (Q1,Q3: 62,160)), and two patients were stable at the end of the pediatric follow-up, but presented a relapse in adulthood. Among the 18 patients retrospectively registered in adulthood, only three (17%) were healed during childhood, with no relapse during the adult follow-up (median duration 87 months range (35–204 months)), and four (22%) were stable at the end of the pediatric follow-up, but presented a relapse in adulthood. Taking into account the whole series (Figure 2), at the end of pediatric follow up, half of the patients were stable (no activity of the disease while without treatment); the other 26 (50%) patients still required a treatment. Among the stable patients, five (19%) presented relapses in adulthood. Among the patients under treatment at the age of the transition of care, only one (3.8%) presented no relapse afterwards and was stable in adulthood.

**Figure 3:**
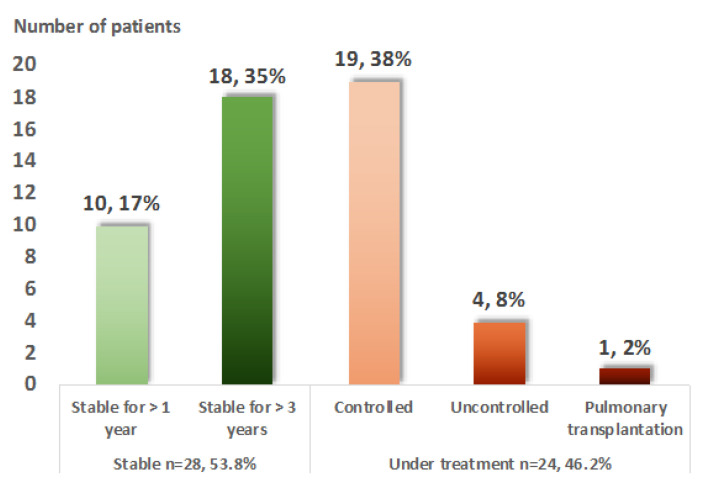
Evolution in adulthood of 52 patients with pediatric-onset sarcoidosis. The adulthood evolution is divided into two almost equal groups: 51.9% of the patients were stable at the last evaluation, whereas 48.1% were still requiring treatment. Five patients (10%) were still uncontrolled under treatment or required lung transplantation.

Finally, at the last adult evaluation, treatment was still required in 28 (53.8%) patients (Figure 3). Among them, only four (7.7%) presented an uncontrolled disease despite treatment. No death was recorded, but one patient required a pulmonary transplantation at the age of 49.

#### 3.2.2. Organ Involvement

Organ involvement and constitutional symptoms were compared between childhood and adulthood in the 30 patients who presented a progressive sarcoidosis in adulthood (i.e., who presented at least one relapse after the age of 18) (Table 2). The median number of involved organs was significantly lower in adulthood with proportionally less lung, liver, spleen, peripheral lymph node involvement, and less patients reporting fever and fatigue (*p* < 0.05).

#### 3.2.3. Relapse During Follow up

**Figure 4:**
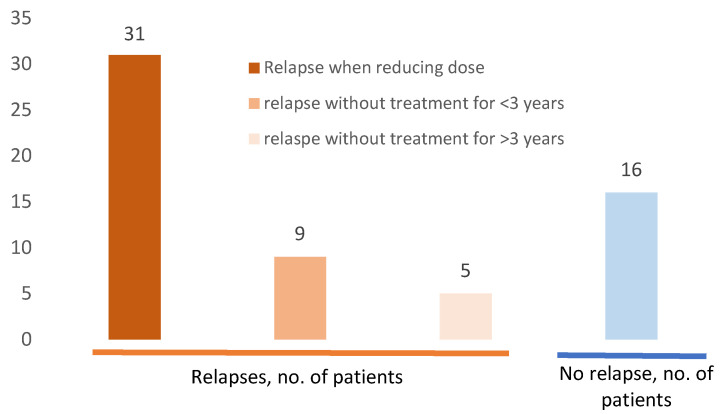
Sarcoidosis relapses among 52 patients with pediatric-onset sarcoidosis. Sixteen (30.7%) of the patients did not present any relapse. Among the 36 (69%) patients who presented relapses, the majority (*n* = 31, 59%); occurred when reducing doses of corticosteroid; nine (17%) after less than three years without any sarcoidosis treatment, and five (9.6%) after more than three years without any treatment. Some patients presented different type of relapses.

Figure 4 reports sarcoidosis relapses among the 52 patients. One hundred and ten relapses were recorded in 36 patients (69.2%), mostly in adulthood (84 relapses; 86%, in 30 patients, 84%). In most cases, relapse occurred following corticosteroid tapering (93 relapses, 84.5%). However, exacerbations/relapses were also described in five patients (seven episodes) after three years of stability without any treatment. Patients who presented relapses only during childhood were few (six patients, 17%), whereas 21 (58%) patients presented relapses only during adulthood, and nine (25%) in both childhood and adulthood. Relapse frequently affected a previously involved organ (65.4% patients, 85.4% of relapses), (Supplementary Table S2).

#### 3.2.4. Evolution of Severe Sarcoidosis during Follow-Up

• Severe lung involvement

**Figure 5:**
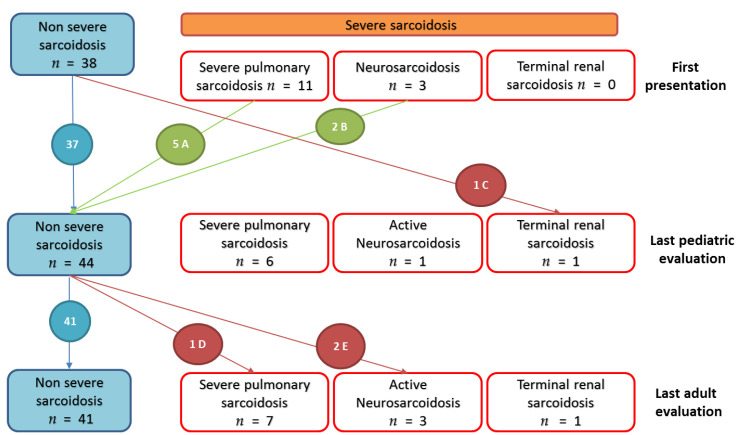
Evolution of severe organ involvements in 52 patients from first pediatric to last adult evaluations. Severe sarcoidosis were defined as: (i) lung involvement with Composite Physiologic index (CPI) > 40 and/or pulmonary hypertension and/or pulmonary fibrosis extent at computed tomography (CT) of more than 20% of the lung [15]; (ii) evidence of central nervous system sarcoidosis requiring a treatment; (iii) heart involvement with spontaneous sustained ventricular tachycardias, arrythmias requiring defibrillator, conduction block requiring pacemaker, heart failure with left ventricular ejection fraction under 40%; (iv) liver involvement requiring a transplantation; (v) kidney involvement requiring chronic dialysis or transplantation. **A**: Five patients with initial severe lung involvement (CPI > 40), improved (normal pulmonary function and thoracic radiography). **B:** Two patients with antehypopituitarism that resolved under treatment, one patient with sequels as facial peripheric palsy and hypopituitarism requiring hormonal supplementation. **C**: one patient with renal, ocular, hepatic sarcoidosis diagnosed at five years old with multiple relapses that led to renal failure at eighteen years old, requiring dialysis. **D**: one patient with subnormal pulmonary function in pediatric follow-up who experiences worsening in adulthood with lung fibrosis and pulmonary aspergillosis. **E**: Two patients with uveitis in pediatric follow-up (and pulmonary involvement) who presented neurosarcoidosis at adulthood evaluation (one and four years after onset of disease, respectively).

Initially, eleven patients had a pulmonary sarcoidosis with severe impaired respiratory function (CPI > 40) but without sign of fibrosis or of pulmonary hypertension. All of them were treated, and five of them improved their respiratory function (normal FVC and DLCO) and radiographic (no abnormality) at the last pediatric evaluation. We noted that two other patients presented a possible severe pulmonary involvement, but which could not be assessed as no DLCO was available. These two patients improved under treatment, one of them showing persistent respiratory function impairment without criteria of severity. Six of the 11 patients with severe pulmonary sarcoidosis displayed persistent severe sarcoidosis until the last adult evaluation, with onset of fibrosis on computed tomography (CT) five to thirteen years after the diagnosis. Two patients presented a particularly severe lung involvement. One patient, without severe presentation at pediatric follow up, showed worsening lung function because of pulmonary fibrosis and chronic pulmonary aspergillosis at the age of 25 (fourteen years after diagnosis). This led to multiple life-threatening hemoptysis requiring six bronchial artery embolizations and right upper lobectomy resection for hemostasis. The other patient presented a precapillary pulmonary hypertension assessed by right heart catheterization at the age of 40, thirty years after diagnosis, followed by multiple lung infections by *Pseudomonas aeruginosa*. He required long-term oxygen therapy and a pulmonary transplantation at the age of 49. (Figure 5 and Supplementary Figure S2).

• Neurosarcoidosis and severe renal involvement

At the pediatric follow up, three patients presented neurosarcoidosis, one patient with epileptic seizure that required antiepileptic therapy and two patients with partial hypopituitarism. These two patients experienced no neurologic relapse under treatment. One patient had no sequelae. The other one required hormone replacement therapy and had persistent facial peripheric palsy. The third patient displayed many neurologic relapses (meningitis, transitory hemiparesis) at adulthood and required biologic agent use. Two other patients presented a de novo neurosarcoidosis at adulthood: meningitis associated with uveitis four years after diagnosis for one, behavioral disorders one year after diagnosis for the other, confirmed by cerebrospinal fluid lymphocytosis. These two patients presented pulmonary and ocular involvement at disease onset. (Figure 5 and Supplementary Figure S2)

Eventually, one patient presented with a renal sarcoidosis diagnosed at the age of 5, with multiple relapses which led to renal failure requiring chronic dialysis since the age of 18 (Figure 5 and Supplementary Figure S2)

• Sequels

Other sequels not fulfilling *stricto sensu* our severity criteria, but handicapping, were also recorded for five patients: chronic renal impairment with creatinine blood level up to 150 µmol/L in two patients, blindness in one patient, bilateral hearing loss for one patient (without alternative neurologic or otologic cause), and one patient presented with hepatic fibrosis and marked chronic cholestasis up ten times normal, without portal hypertension nor sign of hepatic insufficiency. If blindness and bilateral hearing loss are added, up to 13 patients (25%) can be considered as severe with either life-threatening manifestations or a severe disability.

### 3.3. Treatment and Adverse Events

Nearly all of the patients were treated (49, 94%) with corticosteroids (Table 3). We estimated cumulative corticosteroid doses to a median 17,900 mg equivalent prednisone (maximum: 99,000 mg) for a median duration of five years (minimum 4 months, maximum: 32 years). Twenty-five patients were also treated by immunosuppressive treatments including Tumor Necrosis Factor alpha agonists (anti-TNFα): 11 patients had hydroxychloroquine, methotrexate, azathioprine as a corticosteroid-sparing treatment; nine patients had methotrexate, azathioprine, mycophenolate mofetil, or cyclophosphamide for uncontrolled sarcoidosis under corticosteroid therapy, five patients required anti-TNFα because of relapse despite a second-line treatment. At the last evaluation in adult follow-up, 24 (46.2%) patients were still treated with corticosteroid (median of 7.5 mg prednisone equivalent), and 15 (29%) with another immunosuppressive agent. Various adverse events (28 in 18 patients) were reported and were mostly secondary to corticosteroids (notably obesity, diabetes, arterial hypertension, dyslipidemia, reversible amenorrhea). No arteriosclerosis complications (i.e., stroke, myocardial coronary artery disease, nephroangiosclerosis etc.) was recorded. Two (3.8%) patients presented severe complications: one patient presented a bacterial septic shock (*Stenotrophomonas Maltophilia)* secondary to an infection of implantable venous access; another patient experienced a community-based pneumonia (*Staphylococcus aureus)* requiring hospitalization.

### 3.4. Prognostic Factors for Severe Sarcoidosis in Adulthood

We aimed to determine if any of the clinical characteristics of the patients in childhood were associated with severe sarcoidosis in adulthood (*n* = 11) (Supplementary Table S3). Gender, ancestry, age at diagnosis, number of involved organs, Scadding’s chest radiography classification, forced vital capacity, forced expiratory volume in 1 s or lung diffusion factor for carbon monoxide, CPI, or organ involvement were not associated with severe sarcoidosis in adulthood (*p* > 0.05). Similarly, gender, ancestry, age at diagnosis, number of involved organs, Scadding’s chest radiography classification, forced vital capacity, forced expiratory volume in 1 s or lung diffusion factor for carbon monoxide CPI, or organ involvement were not associated with stable sarcoidosis in adulthood (*p* > 0.05), (Supplementary Table S4).

## 4. Discussion

We report here the long-term evolution of childhood-onset sarcoidosis in a large multicentric series. Well-organized networks (RespiRare, RespiFIL) and study groups (GSF, SPLF) for rare lung diseases in France allow close collaborations between pediatric and adult teams. This is a crucial point to promote joint studies, to collect precise phenotypes over time, and to better understand specific subgroups of diseases. Ultimately, patients will benefit from better longitudinal care, avoiding destabilizing gaps between the pediatric and the adult management of their chronic disease.

This original study highlights strong and novel messages: (i) child-onset sarcoidosis is characterized by a multivisceral involvement, a protracted course of the disease, and a need for pursuing follow-up at adulthood in at least half of the patients; (ii) a prolonged treatment is needed, to be counted in years, and half of the patients will still require a treatment in adulthood; (iii) despite very high doses of treatment, especially corticosteroid therapy since childhood, adverse events were less important than expected; (iv) child-onset sarcoidosis is characterized in adulthood by a significant proportion of patients harboring severe lung, neurologic, or renal manifestations: there were 14 patients with a severe disease at onset (33%), eight patients at last pediatric visit (15%) and 11 patients at adulthood (21%). If severe disability is added to life-threatening manifestations, this number goes up to 13 patients in adulthood (25%); (v) sarcoidosis severity cannot be predicted at onset during childhood; (vi) no death was recorded in this study, but mortality is probably underestimated since half of the patients have persistent disease, and for some a severe sarcoidosis, at last follow-up.

### 4.1. Pediatric Sarcoidosis: A Multiorganic Disease

As previously reported in 41 patients in France, but also in 48 patients in Denmark and 27 patients in the United States, sarcoidosis in children is a severe disease with multiorganic involvement (median of 3.4 affected organs in the French series) at disease onset [10,12,13]. It is also characterized by forefront constitutional signs. By contrast, in “classical” adult-onset sarcoidosis, the number of involved organs at diagnosis is often reported to be low and general signs, such as fever and weight loss, are rarely reported [17]). Interestingly, we observed that the number of involved organs in the adulthood evolution of child-onset disease (1.9 per patient) is similar to the one reported in the ACCESS study (approximately below two per patient [7]). Thus, presentation depended more on the actual age of patients than on the age at onset.

Fifty three percent (18/34) of the patients recovered during childhood. Among patients who had persistent sarcoidosis at adulthood, a majority of the patients presented relapses (110 relapses for 36 (69%) patients); with a median of two relapses per patient (range 1–12), most often in adulthood. These relapses occurred most often during adulthood treatment tapering, but were also observed after a long period of clinical stability (over three years), a finding very rare in adult-onset sarcoidosis [18]. Relapses occurred preferentially in a previously affected organ (96, 87.3%), but also in de novo organs (16.4%). The last evaluation at pediatric follow-up thus turned out to be insufficient to predict adult outcome: among the 26 (50%) “stable” pediatric sarcoidosis cases, five (19%) presented relapse during adulthood, including four (15%) patients who presented persistent sarcoidosis requiring treatment. On the contrary, among the other 26 (50%) patients still under treatment at the child-to-adult transition, five (19%) resolved in adulthood. This unpredictable evolution often results in a long duration of the disease (median seven years, range 1.5–44) and prolonged treatment. These results (54% of stability, 46% of persistent disease) are in contrast with the long-term evolution of pediatric sarcoidosis reported by Milman et al. [11]. In this study, after three to 23 years of evolution of the disease (i.e., in 2006), 83% (38/46 patients) recovered, 11% (5/46 patients) were still presenting a chronic disease, and three patients died. However, apart from the 14 years between the present study and Milman’s, the study populations display important differences, notably the patients’ ancestry: all European for the Danish cohort, whereas most of the herein presented patients originated from Africa and the Caribs.

### 4.2. Unexpected Tolerance of Intensive and Prolonged Treatment

As recommended, almost all the patients (49; 94.2%) received corticosteroid therapy as a first-line treatment for a median duration of five (range 1.38–10.3) years. According to pediatric practice for interstitial lung diseases, intravenous pulses were largely used (25; 51%), alone or more frequently in addition with an initial oral corticosteroid therapy [19,20,21]. Moreover, 25 (48%) patients received an immunosuppressive drug for a median duration of three (range 1.1–5) years. In comparison, only 40% of patients with adult-onset sarcoidosis are reported to require long-term treatment [22]. Surprisingly, despite the patients received impressive cumulative dose of corticosteroid therapy (median 17,800 mg, maximum 35,200 mg) and/or prolonged immunosuppressive drugs, the number of patients that underwent adverse events remained limited (18; 35%), and the adverse event was severe in only two (3.8%) patients, which was far less than expected in an adult population [23,24]. Intravenous methylprednisolone pulses are reported to be well tolerated in childhood and are believed to be associated with a low rate of adverse events and less risk for adrenal suppression, even when delivered for years [25,26]. Recommended dose is 10–30 mg/kg/day for three consecutive days at monthly intervals. In children with interstitial lung disease, intravenous methylprednisolone pulses are suggested to be associated with a better tolerance than oral glucocorticoids by several authors Thus, as in the present study, this method is recommended for children with significant interstitial lung disease who are likely to require high-doses of glucocorticoids over a prolonged period of time. Adverse events of glucocorticoids are well recognized in adulthood [23,27]; therefore, cumulative corticosteroid doses should be closely monitored. Corticosteroid-sparing agents should be recommended when the needed daily dose of glucocorticoids exceeds 10 mg/day for a long duration or when adverse events occur. Such policy might help avoiding most relapses following glucocorticoid dose tapering.

Regarding the initial severity of the disease, the risk of long-term relapse and the potential adverse effects of prolonged treatments, a long-term follow-up is highly recommended in patients with pediatric-onset sarcoidosis. This also means that a patient with a stable sarcoidosis reaching teenage or adulthood should probably be considered as being in remission rather than cured, and thus should benefit from a pediatric to adult transition of care.

### 4.3. Severe Pediatric-Onset Sarcoidosis at Adulthood

At adulthood, 11 (21%) patients presented a severe life-threatening sarcoidosis, more than reported in a large adult-onset series (8.5%) [17]. Interestingly, the number of patients with severe sarcoidosis changed from child-onset (14) to last pediatric visit (8) and adulthood (11), with some patients becoming free of severity while other became severe. Parenchymal lung involvement was the main organ involvement associated with severity. In childhood, severity was supported in all cases by a CPI > 40 (composite index calculated from pulmonary function tests (FVC, DLCO, FEV1) which has been proved to predict survival in adult sarcoidosis [15]). In childhood, there was no pulmonary hypertension nor pulmonary fibrosis, and five out of 11 patients with severe pulmonary sarcoidosis improved significantly. In adults, severity associated with pulmonary fibrosis confirmed on CT images was observed in seven patients (13.4%). Pulmonary fibrosis was never observed at diagnosis, only once during childhood evolution (at 15 years-old), and mainly occurring in adulthood. Pulmonary fibrosis is extremely rare in childhood ILD and is poorly reported in children with sarcoidosis [13,28,29,30,31]. In adults with sarcoidosis, evolution towards pulmonary fibrosis has been reported in around 14% [32], about the same proportion as in our cohort. Of importance, five other patients presented a severe respiratory function (defined herein by a CPI > 40) that improved their respiratory function (CPI < 40) with treatment. Two patients had extreme severity related to well-known complications of pulmonary fibrosis: pulmonary aspergillosis, precapillary pulmonary hypertension, and bacterial supra infection [24,33]. Altogether, these findings indicate that the Walsh’s algorithm (defined such as CPI > 40, fibrosis > 20%, and pulmonary hypertension estimation) [15], which accurately predicts a worse evolution, is not always applicable at childhood.

In the present study, three patients displayed a severe neurosarcoidosis, one of them since childhood and two since adulthood. These three patients also presented an initial bilateral uveitis. One patient presented renal involvement since the first presentation, but that led to a terminal renal failure requiring dialysis since the age of 18 despite treatment.

Despite not being life-threatening, severe disability might also be added as severe manifestation. While blindness in one patient was certainly due to sarcoidosis, a sarcoidosis origin could only be considered as probable for the patient with bilateral hearing loss.

### 4.4. Risk Factors for a Severe Evolution of the Disease

In this follow-up study, we searched for clinical risk factors at presentation that could predict the evolution of the disease. In the French pediatric study, only the number of involved organs at diagnosis was associated with a pejorative evolution in terms of relapses. Unfortunately, in this large and long study, no risk factor could be identified [6].

### 4.5. Extreme Severe Evolution May Be Underestimated

One patient required a pulmonary transplantation at the age of 49. No patient died. However, it has to be acknowledged that this transplanted patient as well as one of the most severe patients (with life-threatening hemoptysis secondary to aspergillosis) were the oldest (49 and 55 years old), and displayed the longest disease evolution (39 and 44.5 years). Giving that the median age of the included patients was much younger (23 years) and the median length of follow-up was 11.48 years, this morbidity could be underestimated. A longer follow-up of the patients will provide more accurate information on a potential severe evolution of the disease. We may hypothesize that the seven severe lung sarcoidosis would have a reduced survival expectancy given the lung fibrosis and function lung impairment [15,33,34].

### 4.6. Study Limitations

The large range of the patient age at the time of the study and of the duration of follow-up certainly biased our results. However, to date, the present study describes the largest series of pediatric-onset sarcoidosis ever reported. In order to gather such large study population, we also had to collect retrospective data from 40 years ago, reported by adult medical doctors. A major point is that the present series included two types of inclusions: (1) A prospective one from childhood onset that allowed us to accurately estimate the rate of patients with a still active sarcoidosis in adulthood; (2) A retrospective inclusion of patients recruited in adulthood with a persistent sarcoidosis and who retrospectively reported pediatric onset of the sarcoidosis. The accuracy of such data grouping can be discussed as well as the highly probable under-reporting of resolved pediatric sarcoidosis cases between 1976 and 2000. However, gathering these two inclusion sets allowed us to obtain a larger study population of adult patients with childhood-onset sarcoidosis, which is crucial in such a rare disease. They provided complementary information that, among the variables collected at disease onset, could allow us to predict sarcoidosis severity and treatment status in adulthood. Furthermore, patients were mostly from the Paris area and surrounding regions, therefore under-reporting of other regions is highly possible. Lastly, even if glucocorticoid side effects were collected, failure to thrive (i.e., the gap between the predicted and the current size of the patient) and osteopenia could not be assessed in enough patients to be reported. Nevertheless, we assessed body measurements for 41 patients (21/29 women, 20/23 men) in adulthood. They reached a median of 161 cm for women and 175.5 cm for men, which is close to the reported standard sizes in France (164 cm for 18–29 old women, 178 cm for 18–29 old men [35].

## 5. Conclusions

Pediatric-onset sarcoidosis is a rarely reported condition that often evolves toward a chronic disease in adulthood, with almost half of patients requiring long-term treatment. Patients display a significant proportion of severe manifestations that cannot be predicted in childhood. Interestingly, adverse events related to treatments, mainly glucocorticoids, were less important than expected, despite high cumulative drug doses. Severity and morbidity of this disease should not be underestimated, and argue for a life-long follow up of these patients.

## Figures and Tables

**Table 1:** Clinical characteristic of the 52 included patients.

Clinical Characteristics	Study Population (*n* = 52)
Female, *n* (%)	29 (56%)
Ancestry, *n* (%)	
European	5 (9.6%)
Sub-Saharan Africa/Caribbean	37 (71.15%)
North African	9 (17.3%)
India	1 (1.9%)
Age at diagnosis, in years, mean ± SD (rv)	12.03 ± 2.715 (5; 15)
Age at the last evaluation, in years, median (Q1–Q3)	23.6 (19.25–26.48)
Length of follow-up, in years, median (Q1–Q3)	11.48 (6.48;14.5)
Delayed diagnosed comorbidity, *n* (%)	
Crohn disease	2 (3.8%)
Acquired hemophilia	1 (1.9%)
Immune thrombocytopenia	1 (1.9%)
Hemochromatosis	1 (1.9%)
Idiopathic hemophagocytic lymphohistiocytosis	1 (1.9%)

SD: standard deviation, rv: range values, Q1: first quartile, Q3: third quartile.

**Table 2:** Evolution of organ involvement among the 30 patients who presented an active sarcoidosis in adulthood.

	First Presentation *	Adulthood **	*p*
Organ involvement, median, (Q1–Q3)	4 (2.7–4)	1.5 (1–2)	<0.0001 ^1^
Lung, *n* (%)	27 (90%)	14 (47%)	0.0006 ^2^
Eye, *n* (%)	15 (50%)	11 (36.6%)	ns ^2^
Kidney, *n* (%)	4 (13.3%)	2 (6.6%)	ns ^2^
Liver, *n* (%)	17 (56.3%)	7 (23.3%)	0.017 ^2^
Peripheral lymph node, *n* (%)	13 (43%)	3 (10%)	0.007 ^2^
Joints, *n* (%)	5 (17%)	1 (3.3%)	ns ^2^
Skin, *n* (%)	5 (17%)	3 (10%)	ns ^2^
Spleen, *n* (%)	6 (20%)	1 (3.3%)	ns ^2^
Central neurologic, *n* (%)	3 (10%)	3(10%)	ns ^2^
General signs, *n* (%)	11 (36.7%)	2 (6.6%)	<0.05 ^2^
Fever, *n* (%)	8 (26%)	0 (0%)	0.01 ^2^
Fatigue, *n* (%)	11 (36%)	2 (6.6%)	<0.002 ^2^

* Assessment of organ involvement at the first pediatric evaluation. Q1: first quartile, Q3: third quartile. ** Assessment of organ involvement in adulthood defined as a relapse after the age of 18. ^1^: *p* value obtained by Wilcoxon matched pairs signed rank test, ^2^: *p* value obtained by Fisher’s exact test. ns: nonsignificant.

**Table 3:** Overall treatments and adverse events in the 52 included patients.

	Study Population (*n* = 52)
Treatment during pediatric and adult follow-up	
Corticosteroids therapy, *n* (%)	49 (94.2%)
Cumulative dose, in mg, median (Q1–Q3)	17,900 (9900-35,200)
Duration of treatment, in years, median (Q1–Q3)	5 (1.38-10.3)
Intravenous pulse, *n* (%)	25 (48.1%)
Other immunosuppressive treatment, *n* (%)	25 (48.1%)
Duration of treatment, in years, median (Q1–Q3)	3 (1.1-5)
Reason for the treatment	
Corticosteroid dependence	11 (21.1%)
Resistance to corticosteroid therapy	9 (17.3%)
Biotherapy	5 (9.6%)
Adverse events of treatment, *n* patients (%)	18 (35.3%)
Obesity (BMI > 30 kg/m^2^)	8 (15.4%)
Overweight (BMI > 25 kg/m^2^)	4 (7.7%)
Insulin-dependent diabetes	2 (3.8%)
Arterial hypertension	1 (1.9%)
Dyslipidemia	1 (1.9%)
Chronic glaucoma	2 (3.8%)
Posterior cataract	3 (5.8%)
Depression	2 (3.8%)
Septic shock	1 (1.9%)
Pneumonia	1 (1.9%)
Corticotrope deficiency ^1^	1 (4%)
Reversible amenorrhea ^2^	1 (1.9%)

^1^ Among the patients with no more corticosteroid therapy. ^2^ Secondary to glucocorticoid. Q1: first quartile, Q3: third quartile.

## Supplementary Materials

The following are available online at https://www.mdpi.com/2077-0383/9/7/2097/s1, Table S1: Comparison between prospective pediatric onset sarcoidosis and retrospective reported pediatric onset sarcoidosis, Table S2: Relapses of sarcoidosis in adulthood. Table S3: Comparison between severe and non-severe sarcoidosis patients, Table S4: Comparison between stable and under treatment sarcoidosis patients, Figure S1: Sarcoidosis evolution from last pediatric evaluation to last adult evaluation in the two subgroups of patients, Figure S2: Evolution of severe organ involvements from first pediatric to last adult evaluations.
