# Supplementary material for: Child–Adult Transition in Sarcoidosis: A Series of 52 Patients
Source: J Clin Med. 2020 Jul 3;9(7):2097. doi: 10.3390/jcm9072097 (PMC7408766; doi:10.3390/jcm9072097)
Supplement: Supplementary file 1 [file jcm-09-02097-s001.pdf]

Supplementary Materials for

Child-adult transition in sarcoidosis: a series of 52 patients

Simon Chauveau [1,2], Florence Jeny [1,2], Marie-Emeline Montagne [3], Rola Abou Taam [4], Véronique Houdouin [5], Ulrich Meinzer [6], Christophe Delacourt [4], Ralph Epaud [7], Fleur Cohen [8], Catherine Chapelon-Abric [9], Dominique Israël-Biet [10], Karine Juvin [10], Antoine Dossier [11], Bahram Bodaghi [12], Grégoire Prévot [13], Jean-Marc Naccache [14], Sarah Mattioni [15], Antoine Deschildre [16], Jacques Brouard [17], Abdellatif Tazi [18], Roderich Meckenstock [19], Morgane Didier [1,2], Julien Haroche [8], Annick Clement [3,20], Jean-François Bernaudin [1,2], Hilario Nunes [1,2], Dominique Valeyre [1,2,†], Nadia Nathan [3,20,*,†] and for the French Sarcoidosis Group (GSF) ‡

Supplemental Table S1: **Comparison between prospective pediatric onset sarcoidosis and retrospective reported pediatric onset sarcoidosis**

Clinical characteristic	Prospective pediatric onset sarcoidosis n = 34	Retrospective reported pediatric onset sarcoidosis n =18	p
Woman, n (%)	21 (62%)	8 (44%)	ns
Sub-Saharan Africa / Caribbean Ancestry, n (%)	24 (71%)	13 (87%)	ns
Age at diagnosis, median (Q1-Q3)	12 (10-13.75)	12 (11-14)	ns
Length of follow up, years, median (Q1-Q3)	9.8 (6-13.2)	17.8 (11.7 -30)	0.005
Under treatment at adulthood n (%)	14 (41%)	10 (55%)	ns
Severity at disease onset, n (%)	9 (26.4%)	5 (27.7%)	ns
Severity at end of adult follow up, n (%)	5 (14.7%)	6 (33%)	ns
Organ involved, median (Q1-Q3)	3 (2-4)	4 (2.75-4.25)	ns

Q1: First Quartile, Q3: Third quartile. p value estimated by Mann and Whitney test. ns: Non significant p>0.05

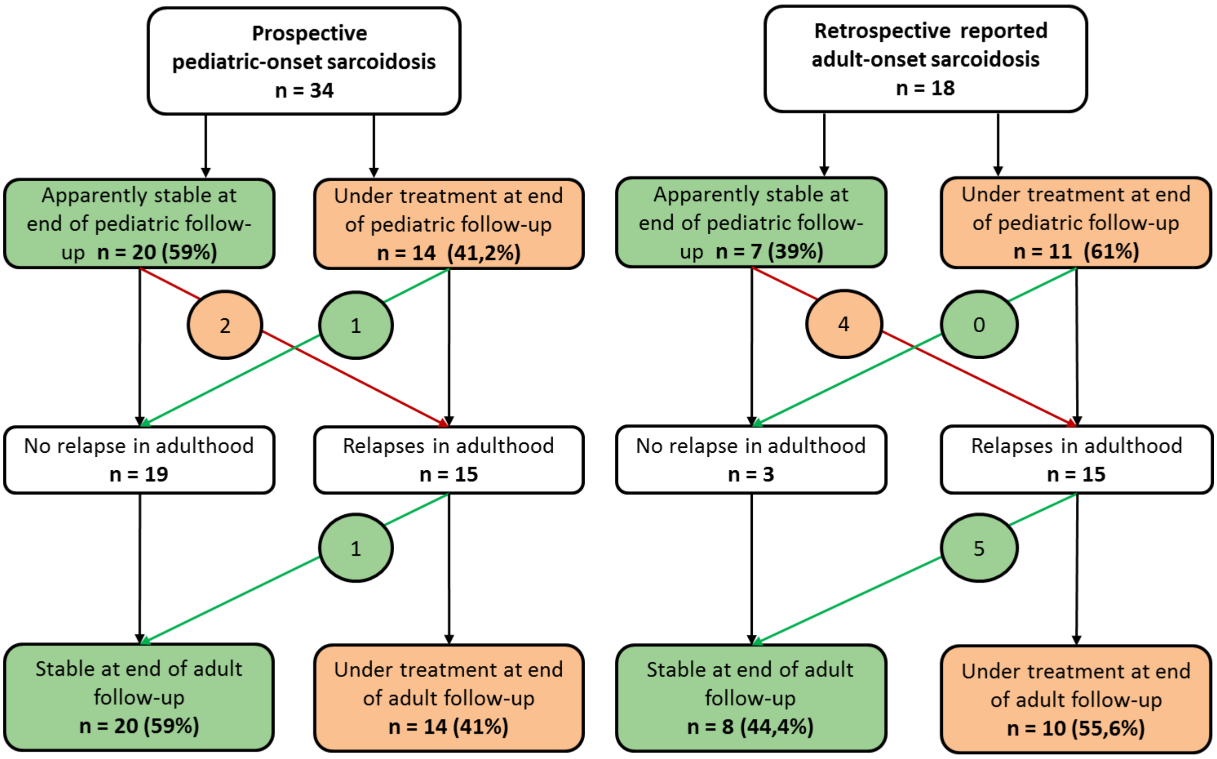

Supplemental Figure S1: Sarcoidosis evolution from last pediatric evaluation to last adult evaluation in the two subgroups of patients. Patients whose evolution changed from "stable" to "under treatment" are circled in orange; patients whose evolution changed from "under treatment" to "stable" are circled in green. (Stable was defined as no relapse more than one year after treatment discontinuation).

Supplemental Table S2: **Relapses of sarcoidosis in adulthood**

	Study population n = 52
**Relapse, patients, n (%) ; relapses, n (%)**	36 (69.2%); 110 (100%)
Relapse when reducing doses	
Patients, n (%)	31 (59%)
Relapse, n (% of total relapse)	93 (84.5%)
Relapse with no treatment for less than 3 years	
Patients, n (%)	9 (17,3%)
Relapse, n (% of total relapse)	10 (9,1%)
Relapse with no treatment for more than 3 years	
Patients, n (%)	5 (9,6%)
Relapse, n (% of total relapse)	7 (6,4%)
Relapse in an initially affected organ	
Patients, n (%)	34 (65.4%)
Relapse, n (% of total relapse)	96 (87.3%) *
Relapse in a different organ than initially affected: "de novo" relapse	
Patients, n (%)	6 (11.5%)
Relapse, n (% of total relapse)	18 (16.4%) *

*Some relapses occurred in different organs, which explain that the sum of relapses exceeds 110.

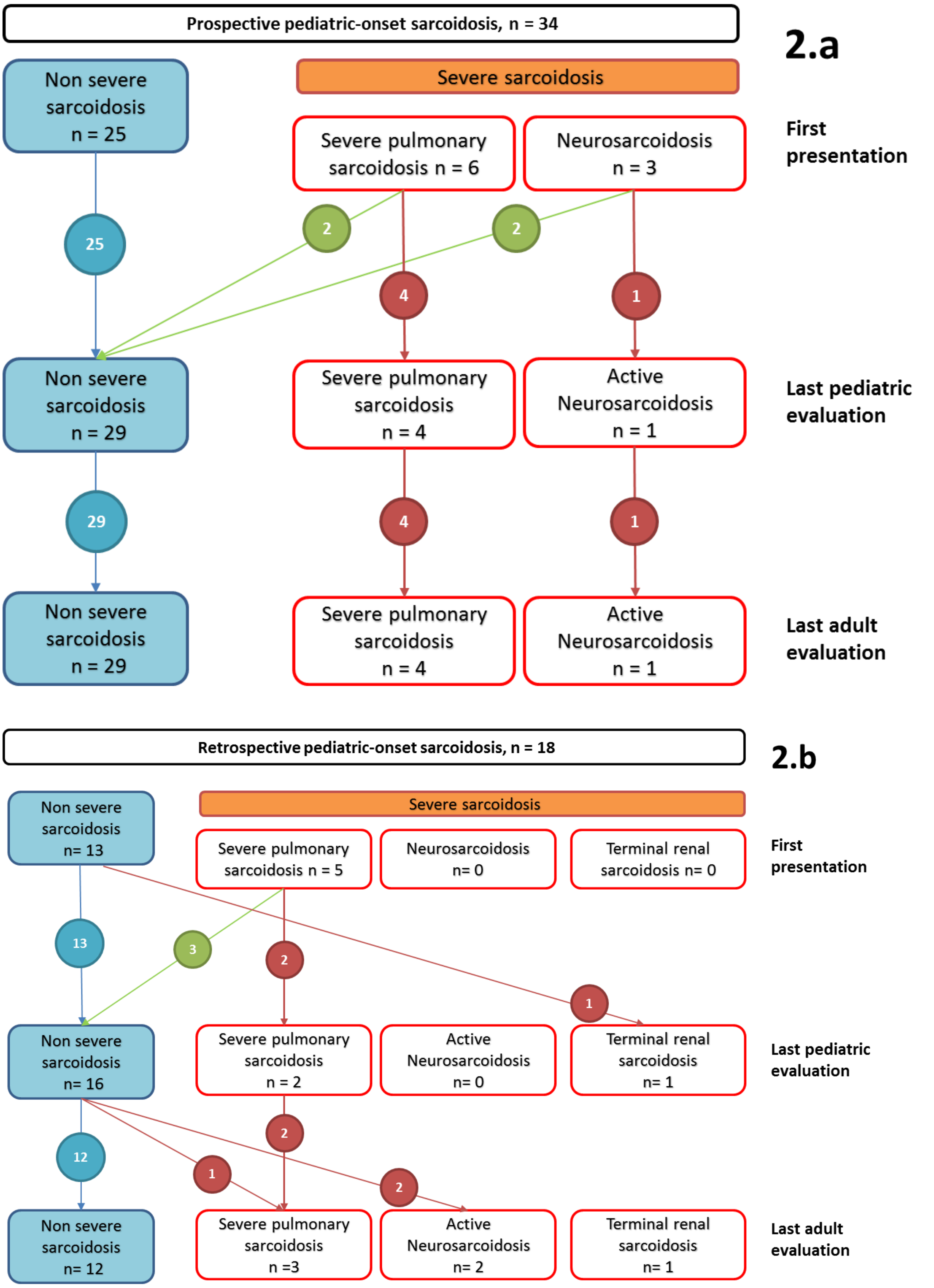

Supplemental Figure S2: Evolution of severe organ involvements from first pediatric to last adult evaluations. 2a: In prospective included pediatric sarcoidosis onset (n=34). 2b: In retrospectively included pediatric sarcoidosis onset (n=18). Severe sarcoidosis were defined as lung involvement with Composite Physiologic index (CPI) > 40 and/or a pulmonary hypertension and/or a pulmonary fibrosis extent of more than 20% of lung [14]; (ii) evidence of heart or central nervous system sarcoidosis requiring a treatment; (iii) liver involvement requiring a transplantation; (iv) kidney involvement requiring chronic dialysis or transplantation.

Supplemental Table S3: Comparison between severe and non-severe sarcoidosis patients

**Clinical characteristics**	Severe sarcoidosis n = 11	Non-severe sarcoidosis n = 41	HR (95%CI)	p
Age at diagnosis, median (Q1;Q3)	12 (11;15)	12(9.5;14)	1.12 (0.9-1.4)	ns
Woman, n (%)	4 (43.4%)	25 (61%)	2.01 (0.6-7.1)	ns
Sub-Saharan Africa or Caribbean ancestry, n (%)	8 (72.7%)	29 (70.7%)	0.78 (0.2-3.9)	ns
**Scadding's chest radiography classification, n (%)**			1.4 (0.8-2.3)	ns
0	2 (18.2%)	19 (46.4%)		
1	1 (9.1%)	4 (9.7%)		
2	5 (45.5%)	6 (22%)		
3	3 (27.3%)	12 (29.3%)		
**Pulmonary function tests**				
FVC % at first evaluation, median (Q1;Q3)	45 (33;80)	68 (58;84)	0.98 (0.9-1.01)	ns
FEV1 % at first evaluation, median (Q1;Q3)	50 (29;81)	80 (59;91)	0.86 (0.22-3.4)	ns
DLCO % at first evaluation, median (Q1;Q3)	75 (60;91)	63 (46;84)	1.1 (0.9-1.2)	ns
Composite Physiologic Index[1]	33 (16;44)	37 (24;53)	0.98 (0.9-1.1)	ns
Organ Involved at diagnosis : median (Q1;Q3)	4 (2.5;4)	3 (2;4)	0.93 (0.58-1.5)	ns
**Organ involvement at diagnosis, n (%)**				
Lung	11 (100%)	34 (83%)	0.95 (0.3-2.9)	ns
Eye	7 (64%)	17 (41%)	2.4 (0.7-8.3)	ns
Kidney	2 (18%)	5 (12%)	2.0 (0.42-9.6)	ns
Hepatic	7 (64%)	19 (46%)	1.2 (0.3-4.3)	ns
Peripheral lymph node	3 (27%)	17 (41%)	0.6 (1.6-2.8)	ns
Skin	3 (27%)	6 (14%)	2.3 (0.6-8.8)	ns
Spleen	1 (9%)	6 (15%)	0.4 (0.05-3.3)	ns
Central neurologic	1 (9%)	2 (5%)	2.2 (0.27-17.9)	ns
Joints	1 (9%)	7 (17%)	0.44 (0.55-3.5)	ns
Fatigue	3 (27%)	18 (44%)	0.78 (0.19-3.1)	ns
Fever	1 (9%)	16 (39%)	0.21(0.03-1.7)	ns
Weight loss	3 (27%)	19 (46%)	0.78 (0.19-3.3)	ns

Q1: First Quartile, Q3: Third quartile. p value estimated by cox regression model. ns: Non significant p>0.05. FVC: Forced Vital capacity, FEV1: Forced expiratory volume in one second. DLCO: Diffusion Capacity for CO. [1]Composite Physiologic Index such as described by Walsh et al, *Lancet Respiratory Medecine*. Severe sarcoidosis was defined such as: (i) lung involvement with Composite Physiologic index (CPI) > 40 and/or pulmonary hypertension and/or pulmonary fibrosis extent at CT of more than 20% of the lung [14]; (ii) evidence of heart or central nervous system sarcoidosis requiring a treatment; (iii) liver involvement requiring a transplantation; (iv) kidney involvement requiring chronic dialysis or transplantation.

Supplemental Table S4: **Comparison between stable and under treatment sarcoidosis patients**

Q1: First Quartile, Q3: Third quartile. p value estimated by cox regression model. ns: Non significant p>0.05. FVC:

**Clinical characteristics**	Stable sarcoidosis n = 28	Under treatment sarcoidosis n = 24	HR (95%CI)	p
Age at diagnosis, median (Q1;Q3)	12.7	12.8	1.13 (0.97-1.3)	ns
Woman, n (%)	15 (55.6%)	14 (56%)	0.49 (0.21-1.1)	ns
Sub-Saharan Africa or Caribbean ancestry, n (%)	19 (70.4%)	18 (72%)	0.76 (0.3-1.9)	ns
**Scadding's chest radiography classification**, n (%)			1.1 (0.7-1.6)	ns
0	11 (39%)	10 (42%)		
1	3 (11%)	2 (8.3%)		
2	5 (18%)	5 (21%)		
3	9 (32%)	6 (25%)		
**Pulmonary function tests**				
FVC % at first evaluation, median (Q1;Q3)	69 (53;85)	68 (66;80)	1.01 (0.99-1.)	ns
FEV1 % at first evaluation, median (Q1;Q3)	80 (59;89)	80 (49;88)	1.0 (0.98-1.02)	ns
DLCO % at first evaluation, median (Q1;Q3)	63 (42;84)	66 (59;86)	1.01 (0.9-1.1)	ns
Composite Physiologic Index	36.5 (24;54)	38 (15;44)	0.98 (0.96-1.01)	ns
Organ Involved at diagnosis: median (Q1;Q3)	3 (2;4)	3.5 (2.75;4)	0.8 (0.5-1.2)	ns
**Organ involvement at diagnosis, n (%)**				
Lung	24 (58.5%)	21 (87.5%)	0.44 (0.1-1.5)	ns
Eye	12 (43%)	12 (50%)	0.97 (0.4-2.2)	ns
Kidney	5 (18%)	2 (8%)	0.77 (0.2-3.3)	ns
Peripheral lymph node	10 (36%)	10 (42%)	1.42 (0.6-3.3)	ns
Skin	6 (21%)	3 (12.5%)	0.24 (0.6-1.1)	ns
Spleen	1 (4%)	6 (25%)	1.6 (0.6-4.3)	ns
Central neurologic	0 (0%)	3 (0.125%)	1.64 (0.5-5.9)	ns
Joints	6 (21%)	2 (8.3%)	0.54 (0.1-2.3)	ns
Fatigue	12 (43%)	9 (37.5%)	1.4 (0.6-3.3)	ns
Fever	11 (39%)	6 (25%)	0.6 (0.2-1.8)	ns
Weight	14 (50%)	8 (33%)	0.98 (0.9-1.01)	ns

Forced Vital capacity, FEV1: Forced expiratory volume in one second. DLCO: Diffusion Capacity for CO. [1]Composite Physiologic Index such as described by Walsh et al, *Lancet Respiratory Medecine*. Stable sarcoidosis was defined as no relapse in more than one year after treatment discontinuation.
